# The Effect of Social Parasitism by *Polyergus breviceps* on the Nestmate Recognition System of Its Host, *Formica altipetens*

**DOI:** 10.1371/journal.pone.0147498

**Published:** 2016-02-03

**Authors:** Candice W. Torres, Neil D. Tsutsui

**Affiliations:** Department of Environmental Science, Policy, and Management, University of California, Berkeley, California, United States of America; University of Arizona, UNITED STATES

## Abstract

Highly social ants, bees and wasps employ sophisticated recognition systems to identify colony members and deny foreign individuals access to their nest. For ants, cuticular hydrocarbons serve as the labels used to ascertain nest membership. Social parasites, however, are capable of breaking the recognition code so that they can thrive unopposed within the colonies of their hosts. Here we examine the influence of the socially parasitic slave-making ant, *Polyergus breviceps* on the nestmate recognition system of its slaves, *Formica altipetens*. We compared the chemical, genetic, and behavioral characteristics of colonies of enslaved and free-living *F*. *altipetens*. We found that enslaved *Formica* colonies were more genetically and chemically diverse than their free-living counterparts. These differences are likely caused by the hallmark of slave-making ant ecology: seasonal raids in which pupa are stolen from several adjacent host colonies. The different social environments of enslaved and free-living *Formica* appear to affect their recognition behaviors: enslaved *Formica* workers were less aggressive towards non-nestmates than were free-living *Formica*. Our findings indicate that parasitism by *P*. *breviceps* dramatically alters both the chemical and genetic context in which their kidnapped hosts develop, leading to changes in how they recognize nestmates.

## Introduction

Eusocial insects display highly cooperative, altruistic behavior in which sterile or effectively sterile workers collectively contribute to brood care, nest maintenance, foraging, and colony defense. To maintain colony integrity, social insects have evolved sophisticated recognition systems that allow them to distinguish nestmates (often kin) from non-nestmates [1, 2]. Individuals recognized as foreign are excluded from the colony, frequently via aggressive encounters [3]. This discriminatory ability is achieved through the detection of chemical cues, or "labels", expressed on the cuticle of insects [4]. These chemical cues are compared to a learned template of referential cues to determine whether an individual should be accepted into the colony [5, 6]. Currently, the most widely accepted model of nestmate recognition asserts that if social insects encounter individuals whose cues do not match their template, that individual is rejected from the colony (see: [7] for review). However, it is likely that an exact match between templates and labels is not necessary for acceptance to occur [7], and highly accurate recognition can be achieved by colonies even when colony members are individually poor at recognizers [8, 9]. Although it has been suggested that the peripheral nervous system acts as a filter for the perception of recognition cues [10, 11], this idea still remains controversial [12].

Cuticular hydrocarbons (CHCs) have long been implicated as the chemical cues used for nestmate recognition [4] and recent literature strongly supports this in ants [13–22]. Cuticular hydrocarbons (CHCs) are waxy, exoskeletal compounds that originally evolved for desiccation and microbial resistance but have since taken on secondary functions as nestmate recognition cues [4, 23–25]. In many insects, cuticular hydrocarbon profiles are species-specific [22, 26]. Often, however, the relative proportions of CHCs vary intraspecifically, as different colonies express distinctive mixtures of these CHCs [7, 27–29]. Although CHCs have a strong genetic component to them [27, 30–32], the complete chemical profile possessed by an individual ant may be modified to include CHCs acquired from colonymates through social interactions including grooming and food exchange, [33, 34] or environmental influences [35, 36].

The large body of research on social insect recognition systems allows us to make predictions about how genetic diversity should affect individual behaviors [2, 6, 7, 31, 37–41]. On average, individuals from high-diversity colonies will possess a larger number of odor cues relative to individuals from low-diversity colonies. This asymmetry in odor diversity should result in more frequent recognition and rejection of individuals from high-diversity colonies. Similarly, individuals from high-diversity colonies are predicted to display less stringent rejection behavior, since they will have imprinted on (and thus accept) a wider diversity of colony odor cues. This asymmetry in template formation is also predicted to produce asymmetries in aggression, with ant from low-diversity colonies more often acting as the aggressors during encounters with ants from high-diversity colonies. Indeed, this type of correlation between asymmetries in diversity and asymmetries in aggression have been recorded between colonies of Argentine ants [41], between monogyne vs. polygyne colonies (e.g. [39, 42–45]), and artificial mixed-species colonies of ants in the lab [5, 46].

Slave-making ants in the genus *Polyergus* are obligate social parasites that depend entirely on their host (genus *Formica*) for nest maintenance, brood care, and foraging. A colony is founded when a mated *Polyergus* queen infiltrates a host nest, kills the *Formica* queen, obtains the queen’s chemical scent, and takes her place as the new queen of the colony [47]. After usurpation, the host workers rear the *Polyergus* queen’s offspring: slave-making workers. With the host queen eliminated, *Polyergus* workers must replenish their supply of slaves by kidnapping pupae from neighboring *Formica* colonies [48–51]. Previous studies of *P*. *breviceps* show that they raid from several different *Formica* colonies annually [52, 53]. As a result, ants from different *Formica* colonies (all of the same species) coexist within the same nest as the *P*. *breviceps* workers and queen.

Slave-making ants appear to take advantage of the fact that their kidnapped host workers, after eclosion, imprint on the chemical cues of individuals they encounter. They then use this information as a reference for nestmate-specific odor cues later in life, resulting in the integration of enslaved workers into the slave-maker colony [54, 55]. Additionally, all colony members may share and recognize a common odor or set of cues, known as a “gestalt” odor [56]. Such a phenomenon may also explain how *Polyergus* colonies maintain cohesiveness among a worker population that originates from different genera and colonies. However, the dramatically different social environments experienced by enslaved and free-living *Formica* provide an opportunity to test hypotheses regarding the impact of diversity (genetic and chemical) on the expression of nestmate recognition behavior.

In this study, we explore the effect of social parasitism by the slave-making ant, *Polyergus breviceps*, on the nestmate recognition of its host, *Formica altipetens*, in eastern-central Arizona. *F*. *altipetens* is a mound-building ant that is primarily single-nested (monodomous) and a has spatial nest distribution suggesting strong intraspecific competition among colonies [57]. Therefore, *F*. *altipetens* are expected to maintain distinct colonies boundaries, presumably via recognition of genetically and chemically similar colonymates and rejection of dissimilar, foreign individuals. However, when *F*. *altipetens* individuals are enslaved by *P*. *breviceps*, we expect the genetic and chemical diversity of the *F*. *altipetens* population within enslaved colonies to increase, as a consequence of *P*. *breviceps* raiding from multiple different *F*. *altipetens* colonies. Thus, when *F*. *altipetens* workers are stolen as pupae and reared in a *P*. *breviceps* colony, they should be exposed to an unusually wide breadth of chemical labels (from enslaved *F*. *altipetens* originating from multiple neighboring colonies and from the slave-makers themselves).

Here we test three hypotheses for how the structure and function of the nestmate recognition system of enslaved *F*. *altipetens* differs from free-living *F*. *altipetens*. We predict that: 1) within-colony genetic diversity will be higher among enslaved *F*. *altipetens* than within free-living *F*. *altipetens* colonies, 2) the composition of chemical cues among enslaved *F*. *altipetens* within colonies will be more diverse than within free-living colonies, and 3) enslaved *F*. *altipetens* will be less aggressive towards non-nestmates compared to their free-living counterparts. We discuss the results of our study in the context of how nestmate recognition may function in *F*. *altipetens*, particularly in the face of social parasitism by *P*. *breviceps*.

## Materials and Methods

We collected individuals for the behavioral portion of this study in July 2008 from the alpine region of Williams Valley, Arizona (elevation: 2650m) where mound-building *Formica altipetens* are enslaved by *Polyergus breviceps* [58]. Based on behavioral assays between free-living *F*. *altipetens* workers and observations in the field, we determined these colonies were monodomous (single-nested) and had no apparent associated satellite colonies (as found in [57]).

In June 2009, we returned to the same field site to collect *F*. *altipetens* and *P*. *breviceps* for chemical and genetic analysis. Additionally, we observed several slave-raids during June 2009 not witnessed in July 2008. This suggests raiding behavior likely occurs earlier and within a shorter time frame at our field site compared to previous records at other locations in Arizona and the western United States (typically July-August; see [52, 53]). To our knowledge, this is the first published record of raiding behavior for this population of *P*. *breviceps*.

### Genetic analysis

Whole genomic DNA was extracted from the heads of chemically analyzed individuals (190 enslaved and 190 free-living *F*. *altipetens*, see the next section for details) using the Qiagen DNeasy Blood and Tissue kit, following the manufacturer’s protocol. We also performed genetic analysis on a subset of individuals collected from 2008.

To determine whether there was higher genetic variation within nests containing enslaved *F*. *altipetens* compared to free-living *F*. *altipetens*, we genotyped worker ants at 11 microsatellite loci developed from three species of *Formica*: Fy4, Fy5, Fy7, Fy13 (F. yessensis; [59]) FL12, FL20, FL21, FL29 (F. lugubris; [60]), and FE16, FE21, FE37 (F. exsecta; [61]). For all loci, we performed 10ul Polymerase Chain Reactions (PCR) using 1.0ul of DNA template, 1X reaction buffer, 300μM of each dNTP, 0.8μM of each primer and 0.04–0.075 units of Promega GoTaq Flexi DNA Polymerase (San Luis Obispo, CA, USA). We used the following temperature program: initial denaturation of 5 minutes at 95°C, 36 cycles of 30 secs at 30°C, 30 secs at the appropriate annealing temperature, and a 30 second extension at 72°C, and a final extension step for 5 minutes at 72°C. Details of the PCR reaction conditions used for each set of loci (Fy, FL and FE) can be found in Table 1. All primers were fluorescently labeled using Applied Biosystems (ABI; Carlsbad, CA, USA) dyes VIC, 6FAM, PET and NED and the size standard LIZ was added to the resulting PCR products. We estimated allele sizes using an ABI 96 capillary 3730xl DNA Analyzer and visualized and scored allele sizes with Peak Scanner v 1.0 (ABI).

**Table 1:** Variations in PCR conditions microsatellite loci using 10ul reaction volumes.

Species of origin and loci	MgCl_2_	BSA^1^	Taq	T_a_^2^
*F*. *yessensis* (Fy4, Fy5, Fy7, Fy13)	1.25 mM	0 mg/ml	0.04 units	48°C
*F*. *lugubris* (FL12, FL20, FL21, FL29)	1.25 mM	0 mg/ml	0.075 units	54°C
*F*. *exsecta* (Fe16, Fe21, Fe37)	1.5 mM	0.2mg/ml	0.075 units	56°C

^1^Bovine Serum Albumin (New England Biolabs), added as a reaction enhancer

^2^Annealing temperature

We determined allele frequency statistics and standard diversity measurements for the colonies sampled using the program GENALEX (v 6.41; [62]). Additional analyses on the microsatellite data (e.g. scoring errors, test for Hardy-Weinberg Equilibrium, linkage disequilibrium) can be found along with the results in (S1 Methods and Results). While these analyses provide additional information about the genetic structure of the colonies, the data gathered did not directly address our genetic diversity hypothesis. We tested for differences in the genetic composition within colonies (number of alleles, expected heterozygosity, etc.) found within enslaved versus free-living colonies using t-tests accounting for unequal variance between samples if necessary or using Mann-Whitney U tests if data was non-normally distributed.

### Chemical data collection and analysis

#### Chemical extraction

In 2009 we collected 19 *P*. *breviceps* and 19 *F*. *altipetens* workers from each of the 10 *P*. *breviceps* colonies and 10 free-living *F*. *altipetens* colonies sampled (190 *P*. *breviceps* workers, 190 enslaved *F*. *altipetens*, and 190 free-living *F*. *altipetens*). We performed whole body extractions by soaking each ant in 200ul hexane for 10 minutes and evaporating the hexane. Each ant was transferred to a vial with 95% ethanol for subsequent genetic analysis (see above).

#### GC/MS analysis

We analyzed ant cuticular hydrocarbon (CHC) profiles using an Agilent 7890A Gas Chromatograph (GC) interfaced with a 5975C Mass Spectrometer (MS) with triple access detector. We used an Agilent DB-5, 30m x 320μm x 0.24μm capillary column for separation of chemical compounds in the GC. To help characterize, compare, and identify the possible components of individual worker profiles, we first analyzed extracts from pooled enslaved *F*. *altipetens*, pooled free-living *F*. *altipetens*, and pooled *P*. *breviceps* workers.

For analysis of individual ant CHC profiles, dried chemical extracts were re-eluted in 60ul of hexane, transferred into a vial insert (Varian #392611594) and 2ul of the extract was injected into the GC/MS by autosampler (Agilent 7683 Series) and run in splitless mode using the following temperature program: 70°C for 2min, 30°C/min to 200°C, 3°C/min to 300°C with a 5 minute hold at the final temperature. We set the MS to perform a full scan from 45–500 amu. A 10% solution of alkane standard mix (Fluka Analytical, MO, USA) was run every 20 samples to monitor any changes in retention times and to provide another method for identifying chemical peaks across sample batches.

#### Determination of chemical composition and variability

We used MSD Productivity ChemStation (rev E.02.00) to detect, identify, and integrate peaks within individual and pooled chemical profiles. To determine whether there was higher variation in the chemical profiles of colonies with enslaved *F*. *altipetens* compared to those of free-living *F*. *altipetens*, we examined individual chemical profiles using two different methods. First, we identified and compared the relative abundance of 14 chemical peaks representing the majority of a subset of the peaks found across all *F*. *altipetens* profiles (Table 2). These peaks could be clearly identified by their mass spectra for all *F*. *altipetens* samples analyzed and, therefore consistently compared across spectra. Second, we counted the total number of peaks in the CHC profiles detected by the ChemStation software meeting particular cut off criteria (see below). This second method allowed us to incorporate analysis of minor peaks contributing less than 3% of the total composition of the individual’s chemical profile. Although these minor peaks were not clearly identifiable by mass spectrum, they may still include cues important for nestmate recognition. For both methods of chemical variation analyses, we only analyzed chemical profiles that had sufficient concentrations of the 14 aforementioned chemical peaks to increase accuracy of detection of chemical peaks in individual CHC profiles and avoid analysis of chemical impurities.

**Table 2:** A subset of fourteen major peaks present in enslaved and free-living *Formica altipetens* chemical profiles and used in the NMDS and ANOSIM analyses. Peak numbers correspond to those found in Fig 1.

Peak Number	Retention time (min)	Suspected chemical class	Diagnostic ions (additional ion^1^)	Present in *P*. *breviceps*
1	12.40	C21 alkane	296	X
2	15.30	C23 monoene	(294) 322	X
3	15.78	C23 alkane	324	X
4	17.68	C24 alkane	338	X
5	19.14	C25 dialkene	(250) 348	
6	19.24	C25 monoene	350	X
7	19.37	C25 monoene	350	
8	19.75	C25 alkane	352	X
9	20.02	C25 dialkene	348	X
10	21.28	C26 monoene	337, 364	
11	23.57	C27 monoene	(350) 378	X
12	23.96	C27 alkane	380	
13	27.66	C29 monoene	(380) 406	X
14	28.15	C29 alkane	408	X

^1^ These peaks, though diagnostic for proper identification of peaks used in this study, were not taken into account when determining the chemical class indicated above.

For our first method, we choose 14 chemical peaks we could consistently detect, identify, and integrate across all individual samples (Table 2). We identified these peaks by analyzing the mass spectra of pooled samples from enslaved *F*. *altipetens* and pooled samples of free-living *F*. *altipetens*. Using the pooled sample data, we built a custom library of chemical peaks that each represented 3% or more of the total chemical profile (below this threshold, peaks could not be reliably detected, even if present). Each library entry contained a chemical peak with its full mass spectrum. Chemical peaks found in individual GC/MS profiles could then be compared to all entries in the library to determine if they match. We analyzed the presence and abundance of these 14 peaks using the library search function in ChemStation and the RTE integrator, respectively. Default parameters were used for the library search strategy except the U+A (set to 3) and the flag threshold (set to 2). We quantified the relative amounts of the 14 chemical peaks identified by dividing the area of each integrated peak by the total area of all 14 peaks used in the analysis. Default parameters were used in the RTE integrator except the minimum peak area detected was set to 0.5% of the largest peak.

For our second variation measurement, we set the ChemStation software to detect peaks within a retention time window of 10–30 minutes using the ChemStation integrator function to eliminate counting peaks representing small amounts of impurities found in the hexane and peaks due to column bleed. We set defaults parameters for ChemStation integrator for all except the initial threshold that was set to 15 to maximize counting of clearly defined but minor peaks. Since the ChemStation integrator may pick up peaks that are not cuticular hydrocarbons, we also ran library match analysis (with default parameters) using a library of custom built chemical peaks found both in *P*. *breviceps* and *F*. *altipetens* that include only CHCs. We used t-tests to determine whether the average number of peaks detected within colonies was higher in enslaved versus free-living *F*. *altipetens*.

We pooled samples of *P*. *breviceps* workers to qualitatively compare CHC profiles of social parasite with their hosts. We analyzed the pooled *P*. *breviceps* chemical profiles using the same RTE integrator parameters mentioned above. We built a custom library of identified chemical peaks based on of this pooled profile, and ran a library match search of the *P*. *breviceps*-specific library against one that contained *F*. *altipetens*-specific compounds, to identify matching chemical peaks between the social parasite and its host. These matches were confirmed by visual comparison of the mass spectra for each chemical peak identified by ChemStation as having a match of 99% (the highest match score).

We used the package *vegan* and the function *metaMDS* in the program R (http://cran.r-project.org, v 2.14.0) to run a Nonmetric Multi-Dimensional Scaling (NMDS) analysis on the 14 peak chemical data set (see first method of chemical variation analysis). We plotted the first and second factors of the NMDS to determine if enslaved and free-living *F*. *altipetens* could be distinguished from one another based on differences in the relative proportions of the 14 chemical peaks mentioned above. For better visualization of potential clustering according to enslaved or free-living status, we used the function *ordiellipse* in the package *vegan* to draw standard deviation ellipses and centroid points on the NMDS plot.

To determine whether there was a difference in the variation of individual worker chemical profiles of enslaved versus free-living individuals, we performed an analysis of similarity (ANOSIM) using the package *vegan* in R and the function *anosim*. ANOSIM uses a rank order dissimilarity matrix (such as one derived from a NMDS analysis) to determine whether there is a significant difference between two or more sample groups based on whether there are greater differences between these groups compared to within groups.

Lastly, we compared the average chemical distance of individuals from their colony centroid points using the R package *vegan* and the program betadisper. Again, only the 14 specified chemical peaks were used to build the chemical distance matrix for this analysis. This addresses our second hypothesis that colonies of enslaved *F*. *altipetens* are more chemically diverse than free-living *F*. *altipetens* colonies. If our second hypothesis is supported, the average chemical distance of enslaved *F*. *altipetens* worker profiles from their colony's centroid point should be larger than that of free-living colonies.

### Behavioral assays and analysis

To test for differences in the behavior of enslaved and free-living *F*. *altipetens* towards non-nestmates, we performed assays where we paired three workers from a designated focal colony against three individuals from a foreign colony. Focal *F*. *altipetens* workers were either enslaved *F*. *altipetens* collected from a *P*. *breviceps* colony (n = 6) or workers from nearby free-living *F*. *altipetens* colonies (n = 6) of approximately the same nest diameter. Focal workers were tested in behavioral assays against two categories of foreign workers: 1.) From nests close to the focal nest (less than 40m away and assumed to be within raiding distance of *P*. *breviceps* as observed in 2009; n = 12) or 2.) From nests far from the focal nest (+150m and known to be outside the raiding distance, see [52], n = 12, [53]). For each assay, we paired workers from the focal and foreign colonies and categorized them into four treatment groups (six colony pairings of 10 trials each were performed per treatment group): enslaved versus free-living *F*. *altipetens* from close nests, enslaved versus free-living *F*. *altipetens* from distant nests, free-living *F*. *altipetens* versus free-living from close nests, and free-living *F*. *altipetens* versus free-living from distant nests. The average distance between a focal colony (with an enslaved or free-living status) and a close nest was 35.8m and 226m distance for far nests. There was no significance difference between the average distance of focal nests from close or far nests with respect to enslaved versus free-living focal colonies.

For each assay, three *F*. *altipetens* workers from a focal colony were marked on their gasters with a small dot of acrylic paint, placed in a neutral arena lined with Fluon, and allowed to acclimate. Next, three unmarked workers from a foreign colony were introduced to the neutral arena and the behaviors and identity of the individual that initiated them (focal or foreign) were recorded for three minutes. If the initiator of aggression could not be determined, the aggressive act was counted as mutually aggressive (neither focal or foreign was counted as an aggressor). We recorded the following as aggressive acts: mandible flaring (threat display), leg and antenna pulling, mandible grappling, and gaster flexing (includes attempts to apply formic acid). All behavioral trials were conducted blind so the observer did not know whether pairings involved negative controls or treatments. Negative controls consisted of 10 trials pairing together nestmates from all involved focal colonies (n = 12).

We analyzed behavioral trials based on the presence or absence of aggression in the focal worker (enslaved or free-living) once we first observed an aggressive act. In other words, if the foreign individual initiated aggression, the focal individual was counted as having an absence of aggression. We tested the hypothesis that enslaved *F*. *altipetens* would be less aggressive towards non-nestmates compared to free-living *F*. *altipetens* by running a generalized linear mixed model (GLMM) in the program *R* using the package *lme4* and the function *lmer*. The response variable we used was presence or absence of aggression by the focal worker. The identity of the focal worker (enslaved or free-living) and the distance of the foreign worker (originating from a nest close or far from the focal nest) were used as fixed-factors in the model. To avoid pseudo-replication, we chose nest identity as a random factor in the model. We tested for the interaction between focal worker identity and distance of the foreign nest and also examined several reductive models looking at the effect of distance alone, the effect of worker identity alone, and both added together in the model (not interacting). A model incorporating the identity of the focal worker and distance as interacting terms yielded the best-fit model based on the AIC scores produced from the models involving the aforementioned factors and ANOVA tests on those models.

## Results

### Genetic composition and variation in enslaved vs. free-living *F*. *altipetens*

We successfully genotyped 371 *F*. *altipetens* workers (189 enslaved, 182 free-living) at 11 loci and found a total of 78 alleles (see S1 Methods and Results for additional population genetic data). When comparing the genetic diversity of enslaved and free-living *F*. *altipetens*, we found the average number of alleles (Na), number of effective alleles (Ne), expected heterozygosity (H_exp_), and unbiased heterozygosity were higher in colonies of enslaved *F*. *altipetens* compared to free-living *F*. *altipetens* (Table 3).

**Table 3:** Comparison of average (±SD) genetic composition found within colonies of enslaved (N = 10 colonies; 189 individuals total) and free-living *F*. *altipetens* (N = 10 colonies; 182 individuals total). Na = number of alleles, Ne = effective number of alleles, and H_exp_ = expected heterozygosity and all other statistics listed are averaged over the 11 microsatellite loci used in this study.

	Na	Ne	H_exp_	Unbiased He
**Enslaved *F*. *altipetens***	4.52 ± 0.46	3.04 ± 0.37	0.551 ± 0.31	0.5661 ± 0.32
**Free-living *F*. *altipetens***	2.46 ± 0.41	1.94 ± 0.24	0.404 ± 0.76	0.4157 ± 0.78
**p-values**^1^	<0.0001	<0.0001	<0.0001	<0.0001

^1^ Results from t-tests or Mann-Whitney U tests

### Chemical composition and variation in enslaved and free-living *Formica altipetens* and their social parasites, *Polyergus breviceps*

**Fig 1:**
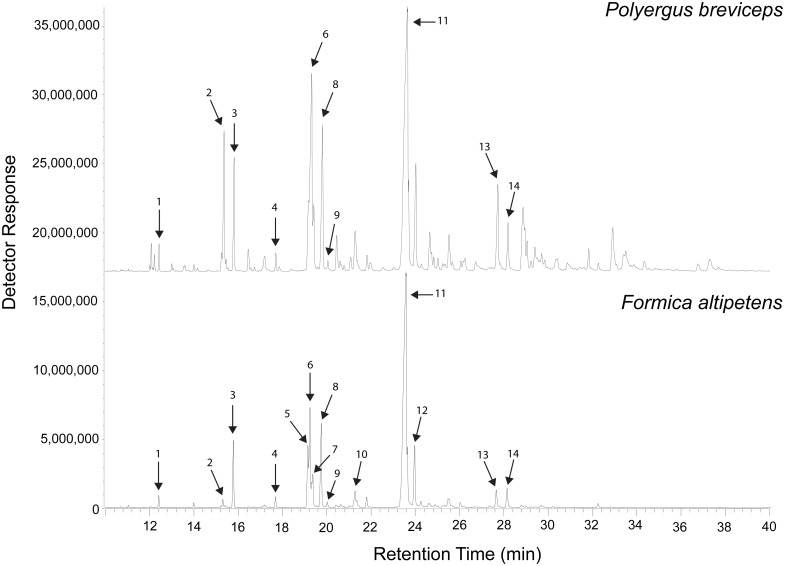
Chemical profiles pooled from 20 *Polyergus breviceps* workers (top) and 20 *Formica altipetens* workers (bottom) from 10 different colonies. Numbered peaks correspond to the chemical components in Table 2.

We found few chemicals were shared between *Polyergus* and their *Formica* slaves. Our comparison of *P*. *breviceps* and *F*. *altipetens* chemical profiles revealed that *P*. *breviceps* have at least twice the number of chemical peaks within their profiles compared to *F*. *altipetens* profiles (Fig 1). We found pooled samples of *P*. *breviceps* had 39 chemical peaks either absent from or below 0.5% of the total peak areas found in the pooled *F*. *altipetens* profiles (70.1% of peaks identified in *P*. *breviceps*). The *F*. *altipetens* profiles shared 16 chemical peaks with those of the pooled *P*. *breviceps* profiles (29.6% of peaks identified in *P*. *breviceps*). Ten of the 14 chemical peaks used in the chemical analysis of individual *F*. *altipetens* profiles matched those in *P*. *breviceps* (Fig 1; Table 2). Overall, it appears the chemical peaks shared between *P*. *breviceps* and *F*. *altipetens* are primarily saturated alkanes and alkenes.

Comparison of pooled samples of enslaved *F*. *altipetens* and pooled samples of free-living *F*. *altipetens* revealed no qualitative differences in peak composition (compare bottom of Fig 1 to S1 Fig).

We analyzed chemical profiles from 326 *F*. *altipetens* workers (146 enslaved, 180 free-living) and looked for differences between enslaved and free-living workers in the quantities of 14 major chemical peaks present (see Table 2) and the total number of major and minor peaks (less than 3% of the total composition of profile peaks).

Qualitatively, there appeared to be no difference in presence of the 14 major peaks for enslaved *F*. *altipetens* compared to free-living *F*. *altipetens* profiles, since all profiles shared these peaks. Quantitatively, however there were differences in the relative proportions of these 14 peaks for enslaved and free-living *F*. *altipetens* profiles analyzed collectively as two groups (instead of by colony). The NMDS plot revealed a subtle distinction between enslaved and free-living *F*. *altipetens* individuals and represented the data well in two dimensions (S2 Fig; converged stress value = 0.072; stress plot R^2^ = 0.99). The ANOSIM showed that the differences among enslaved *F*. *altipetens* individuals were greater than that of free-living *F*. *altipetens* and that, overall, there was a significant difference between the relative proportions of the 14 chemical peaks analyzed for both groups (R = 0.114, p = 0.001). These analyses were completed by assigning individual into enslaved or free-living *F*. *altipetens* groups and comparing them without regard to colony membership. Since these analyses do not address our second hypothesis (individuals within enslaved *F*. *altipetens* colonies are more chemically variable than free-living ones), we compared the average chemical distances of the 14 chemical peaks of enslaved and free-living workers from their colony centroid points to examine chemical diversity. The average (±SD) distance of individual chemical profiles from the centroid of their colony was significantly greater for enslaved *F*. *altipetens* colonies (0.091±0.032) and than for free-living colonies (0.076±0.030)(Mann-Whitney U test, z = 2.05, p = 0.04).

The total number of chemical peaks was significantly higher in colonies of enslaved *F*. *altipetens* than in free-living colonies (mean ± S.D. of enslaved = 53.29± 6.1, mean for free-living = 39.69 ± 4.0, t = 5.91, p<0.001). The same pattern was also true for the number of peaks detected using ChemStation’s library match to find peaks we identified as hydrocarbons (mean of enslaved = 47.61, mean of free = 34.81, t = 5.89, p<0.001).

**Fig 2:**
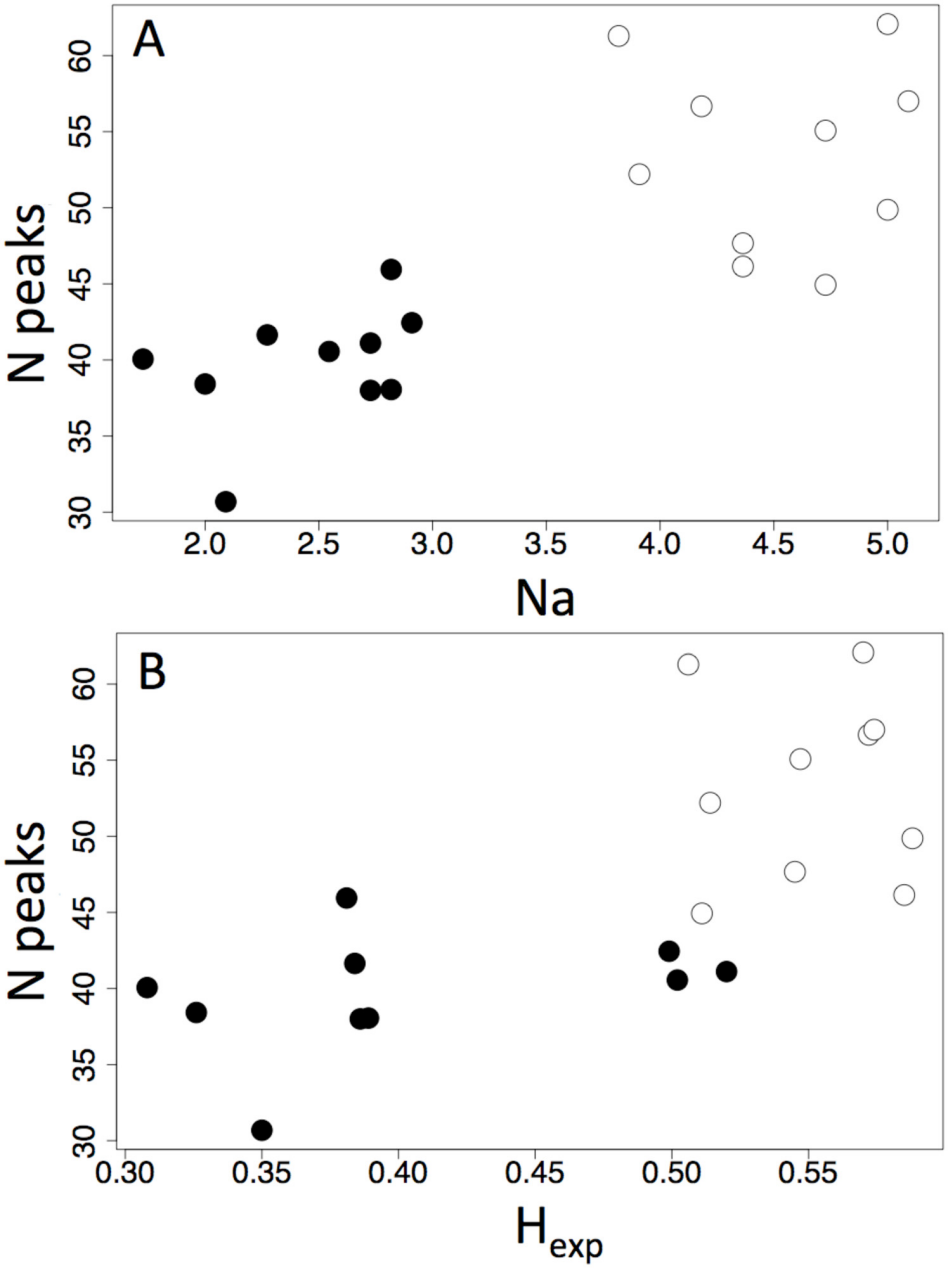
Measurements of genetic diversity plotted against chemical diversity. Average number of alleles (A) and average expected heterozygosity (B) of free-living and enslaved colonies of *F*. *altipetens* (x axes) relative to the average number of chemical peaks found per individual within each colony (y axes). Shaded points represent free-living colonies of *F*. *altipetens* and open points represent colonies with enslaved *F*. *altipetens*. Only individuals for which we acquired both chemical and genetic data from were included in this analysis (146 enslaved and 173 free-living *F*. *altipetens* workers; 10 colonies of enslaved, 10 colonies of free-living)

Considering only those individuals we acquired both genetic and chemical data for, we found colonies with lower average number of alleles (Fig 2A) and expected heterozygosities (Fig 2B) tend to have on average less chemical peaks per individual compared to those with higher measures of genetic diversity and relatively more chemical peaks in their profiles. Free-living *F*. *altipetens* colonies fall mostly in the lower left quadrant of these plots while colonies with enslaved *F*. *altipetens* fall in the upper right.

### Behavior of enslaved and free-living *Formica altipetens* towards free-living non-nestmates

Across all four treatment pairings (n = 24), the average proportion of trials resulting in aggression was 0.795 ± 0.163. Aggression was not observed in any negative control trials.

**Fig 3:**
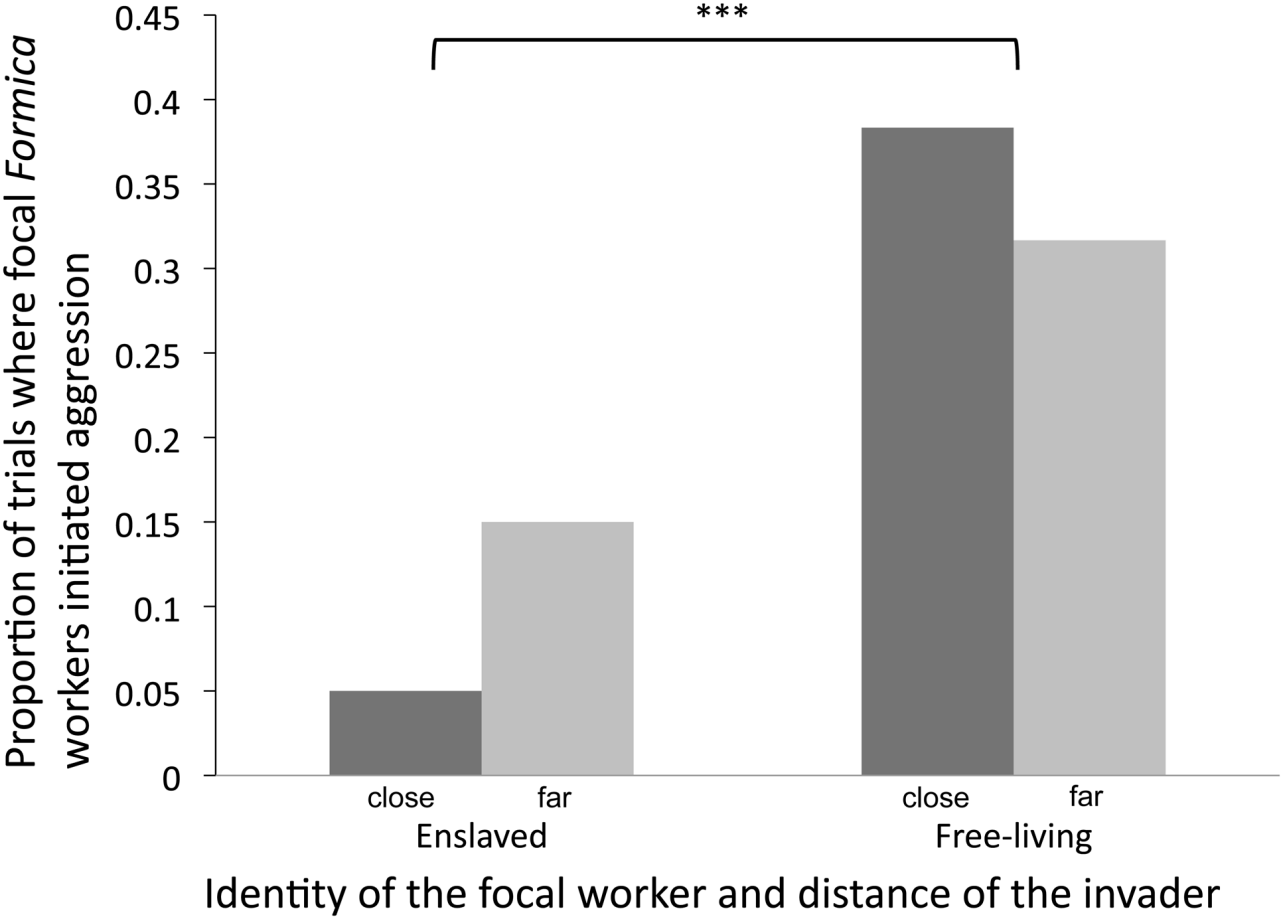
Behavior of enslaved and free-living *Formica altipetens* towards non-nestmates. Y axis displays the proportion of behavioral trials which focal workers (enslaved or free-living) initiated aggression towards non-nestmates from colonies nearby (<40m from focal nest) or far away (+.150m from focal nest). Negative controls between paired nestmates of focal colonies are not shown here as none of these trials resulted in aggression. *** indicates a p-value of < .0005 when comparing enslaved and free-living categories as a whole (lumping close and far variables within).

Overall, enslaved *F*. *altipetens* workers were significantly less aggressive towards free-living non-nestmates compared to workers from free-living focal colonies (Fig 3, z value = 3.61, p = 0.0003). In many cases where enslaved *F*. *altipetens* was not the initiator of aggression (thus designated as having an absence of aggression), the free-living non-nestmate *F*. *altipetens* that it was paired with was the first to initiate aggression and the enslaved ant did not reciprocate with an aggressive response. In contrast, when free-living *F*. *altipetens* were paired with non-nestmates, we noted more mutual acts of aggression (data not shown). Enslaved *F*. *altipetens* displayed a lower frequency of aggression toward non-nestmates from nearby colonies than toward non-nestmates from distant colonies. In contrast, free-living *F*. *altipetens* were less aggressive towards non-nestmates from distant colonies compared those from neighboring ones. However, when we tested the interaction between identity of the focal worker (enslaved versus free-living) and distance of the foreign colony (close versus far from the focal nest), we found it was borderline significant (z value = -1.89, p = 0.057). Distance alone did not appear to determine whether the focal ant was aggressive towards non-nestmates (z value = 1.733, p = 0.08).

## Discussion

Taken as a whole, our data indicate that social parasitism by *Polyergus breviceps* affects the nestmate recognition system of its host, *Formica*. Behaviorally, enslaved *Formica* were less aggressive towards non-nestmates than their free-living counterparts. We propose that this difference in behavior stems from increased chemical and genetic variation among the enslaved workers, thus producing a situation in which nestmate recognition in enslaved *Formica* is altered by the social context of the mixed colony.

Supporting our first hypothesis, we observed significantly higher genetic diversity among enslaved *F*. *altipetens* nestmates compared to free-living *F*. *altipetens* nestmates. This likely stems from *P*. *breviceps* raiding *F*. *altipetens* pupae from several genetically different *F*. *altipetens* colonies. These results are not surprising given our personal observations of raiding behavior in this population of *P*. *breviceps*, and observations from previously studied populations [52, 53].

To test our second hypothesis, we considered 14 of the most prominent and identifiable chemical peaks shared between enslaved and free-living *F*. *altipetens* (i.e. peaks featured in Fig 2; henceforth termed “major" chemical peaks) on a per colony basis and compared the average chemical distances of enslaved and free-living workers from their colony centroid points to compare chemical diversity. We found that enslaved *Formica* workers were significantly more chemically distant, on average, from their colony centroids than were free-living workers. This suggests that there is higher variation in the relative proportions of the 14 major chemical peaks amongst enslaved *F*. *altipetens* colonies compared to those that are free-living.

To further test our second hypothesis, we also counted the total number of chemical peaks detected by the ChemStation software, thus accounting for minor chemical peaks (those with concentrations too low to identify by mass spectrum) in addition to the 14 major peaks identified. This analysis revealed that enslaved *F*. *altipetens* individuals had, on average, a larger total number of chemical peaks within colonies than free-living *F*. *altipetens* colonies.

Analysis of the cuticular hydrocarbon (CHC) profiles of *P*. *breviceps* and enslaved *F*. *altipetens* workers provides additional insights into the chemical cues that may shape the nestmate recognition of enslaved *Formica*. Despite sharing some chemical peaks with their slaves, *P*. *breviceps* appears to maintain its own, genus-specific CHC profile: 39 chemical components were unique to the slave-maker. These results are in line with previous research showing slave-making ants generally maintaining a species-specific profile, despite some matching of cues with their host [63–65]. *Formica altipetens* also had a few chemical peaks that did not appear in the *P*. *breviceps* profile. These unique differences between slave-maker and slave suggest that enslaved *Formica* are exposed to a diversity of chemical cues that are both inter-and intraspecific in origin (whereas free-living *F*. *altipetens* are not).

Since enslaved *Formica* workers are raised in colonies more chemically and genetically diverse than free-living *Formica*, we expected differences in behavior towards non-nestmates. Our behavioral data support the hypothesis that enslaved *Formica* workers are less aggressive towards non-nestmates compared to free-living *Formica*. Few studies have explored colony-mate recognition behavior of the host in a slave-making ant system. Habersetzer [66] showed that free-living *Formica rufibarbis* were always the initiators of aggression when paired with *F*. *rufibaris* enslaved by *Polyergus rufescens*. Enslaved *F*. *rufibarbis* showed no aggression towards enslaved non-nestmate *F*. *rufibarbis* and non-nestmate *P*. *rufescens*. In line with our study, Habersetzer’s findings suggest that parasitism by slave-makers affects the nestmate recognition of their host by reducing aggression towards non-nestmates. However, due to sampling and experimental design, the Habersetzer study could not explicitly test the same behavioral hypothesis we test here (a direct comparison of nestmate recognition between enslaved and free-living *Formica*).

Differences in aggression between enslaved and free-living *F*. *altipetens* could arise from differences in the chemical environment of the two colony types. In particular, enslaved *F*. *altipetens* workers are exposed to the diverse chemical cues of *P*. *breviceps* as well as a diversity of cues from enslaved *F*. *altipetens* from several different colonies. In contrast, free-living *Formica* are only exposed to cues from genetically less diverse (and likely closely related) nestmates. Since chemical cue exposure is key to forming the nestmate recognition template used by ants to distinguish friend from foe [65], template expansion could contribute to the observed reduction in aggression of enslaved *Formica*. Under a template-cue matching model [6], an expanded template that incorporates a larger diversity of cues is expected to produce an overall higher rate of acceptance of encountered individuals. Under this circumstance, we would expect lower rates of aggression towards non-nestmates. Indeed, a previous study using artificially mixed species colonies showed increased chemical dissimilarity among nestmates leads to lower aggression toward ants from other colonies [65]. Similarly, previous studies of the invasive Argentine ant, *Linepithema humile*, have shown that aggression between supercolonies is polarized, with less genetically diverse colonies acting as the aggressors more often than high-diversity colonies [41]. We expected to find lower aggression towards ants from colonies in close proximity to enslaved *Formica* colonies, as those "invaders" are likely to have originated from and/or be closely related to previously raided nests. As such, enslaved *F*. *altipetens* are more likely to be familiar with the chemical cues of neighbors within raiding distance and have these cues in their templates.

Such a model of template broadening also implies that enslaved *F*. *altipetens* need to frequently update their template as new members are added to the colony through seasonal raids. This idea of template updating in response to ecological changes has been previously proposed [67, 68]. For a slave-making ant colony to continue to function as it grows its slave population, template updating seems the likely mechanism for maintaining colony cohesion. In a recently proposed model for nestmate recognition, Esponda and Gordon [9] suggest that overall nestmate recognition is the result of a collection of individual ant decision boundaries in CHC-space that converge upon a single colony-level template. The authors suggest that the decision boundaries of ants can shift as a result of exposure to nestmates or non-nestmates. These boundaries are dictated by individual experience, which can be unique and varied. Given that enslaved *Formica* are all likely to experience a wider diversity of cues upon eclosion that are presumably interpreted as nestmate, their decision boundaries would shift such that more individuals would be accepted as nestmate. Importantly, Esponda and Gordon point out that their model predicts individual chemical profiles do not need to be close to the colony profile in CHC-space for acceptance as a nestmate since an individual ant profiles does not necessarily dictate the boundary between acceptance and rejection. Thus, under this model, enslaved *Formica* can accept *Polyergus* and foreign *Formica* as nestmates even though their profiles may be chemically distant from one another.

A study performed by Lorenzi [29] on the socially parasitic wasp *Polistes atrimandibularis* showed workers in parasitized colonies of *Polistes biglumis* were more tolerant of non-nestmates compared to unparasitized ones. However, unlike our findings (no aggression between pairs of nestmates), Lorenzi found wasp nestmates were also more often rejected in parasitized colonies. Lorenzi reasoned that template broadening in parasitized wasps may make it more difficult for them to compare their template to the cues of other wasps. She concluded that changes in the parasitized wasps' template resulted in a general impairment of their recognition system. While this may also be the case in our system, there may be an additional explanation for the behavioral patterns we saw in parasitized *Formica altipetens*.

Even though enslaved *Formica* displayed less frequent aggression toward free-living *Formica* from nearby colonies (consistent with template broadening as discussed above), we found that they were also less often aggressive toward non-nestmates from distant nests. Because these non-nestmates originated from colonies well outside the raiding distance, the enslaved *Formica* would not have encountered and/or incorporated the cues of such distant non-nestmates into their template. In this situation, reduced aggression could be explained by a shift in the "acceptance threshold" of an individual so that cost of rejecting nestmates with diverse cues is minimized (see [69] for review). When such a shift occurs, mismatches between the template and encountered chemical cues may be tolerated as long as the level of incongruence between the two does not exceed this threshold. Reeve’s response threshold model makes predictions about whether such a threshold will shift, and in what direction, depending on what is the most adaptive situation [40]. In our study system, enslaved *Formica* may shift their acceptance threshold such that the rules of matching the template and the label are less stringent. Indeed, since there is no adaptive benefit for enslaved ants to display stringent nestmate recognition behavior, it is possible these individuals are simply less motivated to display aggression. This would allow enslaved *Formica* to be more permissive of deviations from encountered cues from their template, thereby decreasing overall aggression towards non-nestmates even if they have not encountered them before.

Template broadening and threshold shifts are not mutually exclusive explanations for how increased chemical diversity may lead to a change in the nestmate recognition system of parasitized *F*. *altipetens*. An alternative explanation for the reduced aggression in enslaved *F*. *altipetens* is that *P*. *breviceps* could choose to raid (or be more successful raiders of) host colonies that are intrinsically less aggressive. In this case, parasitism by *P*. *breviceps* could provide selection for higher aggression in *F*. *altipetens* so that members from free-living host colonies are more aggressive relative to those that have been parasitized. Such selection for aggression in host colonies has been found in another North American slave-making ant system where *Protomagnatus americanus* parasitizes *Temnothorax longispinosus* [70].

Our study contrasts with other studies that compared the nestmate recognition systems of monogynous (single queen) with those of polygynous colonies (multiple queens). Martin *et al*. [71] found that *Formica execta* colonies with higher genetic diversity (polygynous) had reduced nestmate recognition cue diversity compared to those that were less genetically diverse (monogynous). Similarly, a recent study of European *Formica fusca* showed that despite higher genetic diversity in colonies with multiple queens, diversity of chemical cues and aggression towards non-nestmates did not differ between monogynous and polygynous conspecifics ([72], also see citations within). Our results may differ from studies comparing monogynous and polygynous species because enslaved *F*. *altipetens* may not possess a gestalt mechanism that would homogenize the colony odor among nestmates. Additionally, the ecological context the produces genetic diversity in our system (slave-raiding) is fundamentally different from that of the mechanism in a polygynous colony (multiple reproducing queens), and we may therefore expect differences in how the nestmate recognition evolves in the two systems.

Regardless of underlying causes for differences in nestmate recognition behavior of enslaved and free-living *F*. *altipetens*, our data clearly suggest *P*. *breviceps* alters the genetic and chemical conditions under which enslaved *F*. *altipetens* are reared. To our knowledge, this is the first study to show how increased genetic and chemical variation caused by slave raiding affects the recognition systems of enslaved ants. Studies such as these allow us to explore possible mechanisms of template broadening and shifts in the threshold of acceptance as they relate to the behavioral response of an organism. These components are important for understanding the perception and action aspects of nestmate recognition in eusocial insects (how individuals evaluate, interpret, and act upon differences in the template of chemical cues encountered). Additionally, the nestmate recognition behavior of *F*. *altipetens* could serve as a model for studies of indirect genetic effects, as the behavioral phenotype of focal workers appear to be determined by the genetic identity of their intraspecific and interspecific nestmates. Finally, this study provides insight into the ecological consequences of social parasitism, which is essential for understanding possible co-evolutionary dynamics between social parasite and their hosts.

## Supporting Information

S1 Methods and Results Additional analyses of microsatellite data collected from enslaved and free-living *Formica* and whole population genetic data and outcome of HWE and LD analyses. (DOC)

S1 Fig Pooled chemical profile of 20 free-living *Formica* workers from 10 different colonies. (TIF)

S2 Fig NMDS (Nonmetric multi-dimensional scaling) plot of chemical profiles from enslaved and free-living *Formica altipetens* workers. Plotted points are based on the relative proportions of 14 chemical peaks detected in cuticular hydrocarbon profiles of individual *F*. *altipetens* workers from enslaved colonies (Ens; red triangles) or free-living colonies (Free; blue circles). To illustrate clustering by either enslaved versus free-living status, standard deviation ellipses with centrioid points are plotted.
(TIF)

S1 Table GPS coordinates of colonies sampled. (XLSX)

S1 Dataset Allele data for individual *Formica* sampled. (XLS)

S2 Dataset Areas for the 14 chemical peaks analyzed across individuals sampled. (XLS)

S3 Dataset Raw behavioral data set including presence/absence of aggression for each trial conducted. (XLSX)
