# Supplementary material for: The Effect of Social Parasitism by Polyergus breviceps on the Nestmate Recognition System of Its Host, Formica altipetens
Source: PLoS One. 2016 Feb 3;11(2):e0147498. doi: 10.1371/journal.pone.0147498 (PMC4740506; doi:10.1371/journal.pone.0147498)
Supplement: S1 Methods and Results — (DOC) [file pone.0147498.s001.doc]

Torres and Tsutsui, 2015

*PLOS One* Submission

**SUPPORTING INFORMATION**

The effect of social parasitism by *Polyergus breviceps* on the nestmate recognition system of its host, *Formica altipetens*

**METHODS**

**Additional analyses of microsatellite data collected from enslaved and free-living *Formica* *altipetens***

We identified scoring errors due to stuttering, null alleles, and large allele dropout across all individuals analyzed using MicroChecker (v 2.2.3, Van Oosterhou*t et a*l. 2004). We used GENALEX (v 6.41; Peakall and Smouse 2006) to test whether loci were in Hardy-Weinberg equilibrium at the colony and whole population levels. The program Arlequin v 3.1 (Excoffie*r et a*l. 2005) was used to test for linkage disequilibrium (LD) across all pairs of loci used in this study. Since LD can often occur because of undetected population structuring (as we might expect given this population could be structured by colonies), we also tested for evidence of LD within each colony and compared the amount of LD found in enslaved versus free-living colonies. We tested for differences in the frequency of loci in HWE and frequency of LD found within enslaved versus free-living colonies using t-tests accounting for unequal variance between samples if necessary or using Mann-Whitney U tests if the data was non-normally distributed.

**RESULTS**

**Whole population genetic data and outcome of HWE and LD analyses**

For all 371 *F. altipetens* workers (189 enslaved, 182 free-living) that we were able to genotype at 11 different loci, we found the average number of alleles per locus was 7.091 ± 1.676 (SE) and the average effective number of alleles was 3.473 ± 0.653. Across the whole population, the expected heterozygosity (He, unbiased) was 0.589± 0.075(SE) and the average fixation index (F­st) was 0.071 ± 0.044(SE).

The test for Hardy-Weinberg Equilibrium (HWE) for the whole population showed only three loci with non-significant departures: Fy5, FL12, and FE21.

Analysis using MicroChecker suggested no evidence of large allele drop out for any of the loci. However, possible null alleles were suggested for Fy4, Fy7, FE16, FL20, and FL29.

Tests for pair-wise linkage disequilibrium between all pairs of loci across all *F. altipetens* individuals revealed that 80% of the possible loci pairs were in linkage disequilibrium.

Since the formation of colonies may affect how populations of ants are structured, we also tested for departures from HWE on a per colony basis. For each colony, we found no loci that were consistently in or out of genetic equilibrium as would be expected since these colonies appear to violate several assumptions of HWE. However, the average number of loci in HWE for enslaved *F. altipetens* colonies was 7.8 ± 1.87 (mean ± SD) while for free-living colonies the average number of loci in HWE was 4.3 ± 1.49 and the difference between these were significant (t = 4.6179, p=0.0001). This pattern suggests that population subdivision is likely a strong contributor to the observed departures from HWE.

Although the average LD on a per colony basis (27.9 ± 12.8%) was lower than if all individuals were treated as one population (see above), we found no significant difference between the percentages of pair-wise LD found in enslaved and free-living *F. altipetens* colonies (t= -0.7728, p= 0.45).

**REFERENCES FOR SUPPORTING INFORMATION**

Excoffier L, Laval G, Schneider S (2005) Arlequin (version 3.0): An integrated software package for population genetics data analysis. *Evolutionary Bioinformatics*, **1**, 47-50.

Van Oosterhout C, Hutchinson WF, Wills DPM, Shipley P (2004) MICRO-CHECKER: software for identifying and correcting genotyping errors in microsatellite data. *Molecular Ecology Notes*, **4**, 535-538.
