# Supplementary figures and images for: The Effect of Social Parasitism by Polyergus breviceps on the Nestmate Recognition System of Its Host, Formica altipetens
Source: PLoS One. 2016 Feb 3;11(2):e0147498. doi: 10.1371/journal.pone.0147498 (PMC4740506; doi:10.1371/journal.pone.0147498)

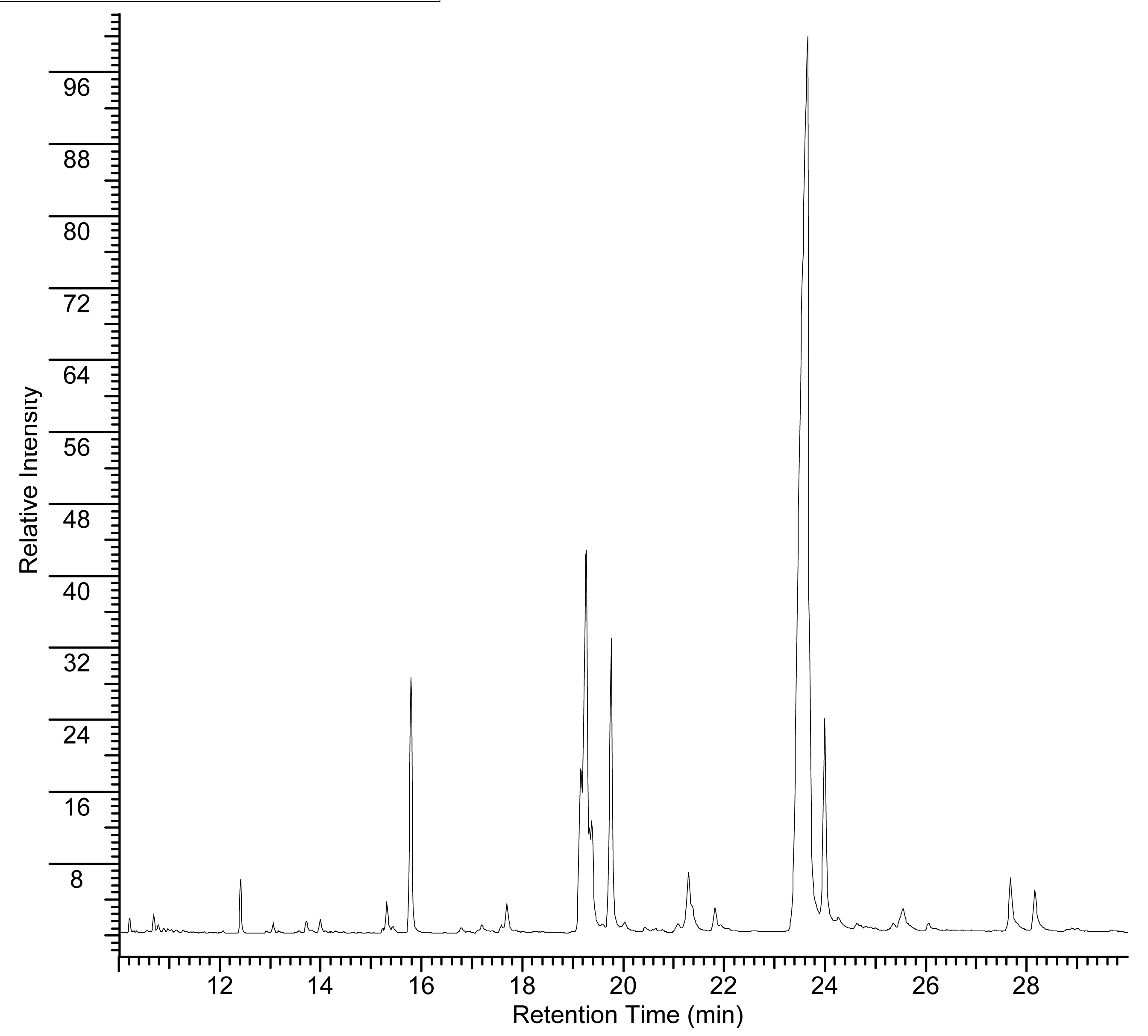

Supplement: S1 Fig — (TIF) [file pone.0147498.s002.tif]

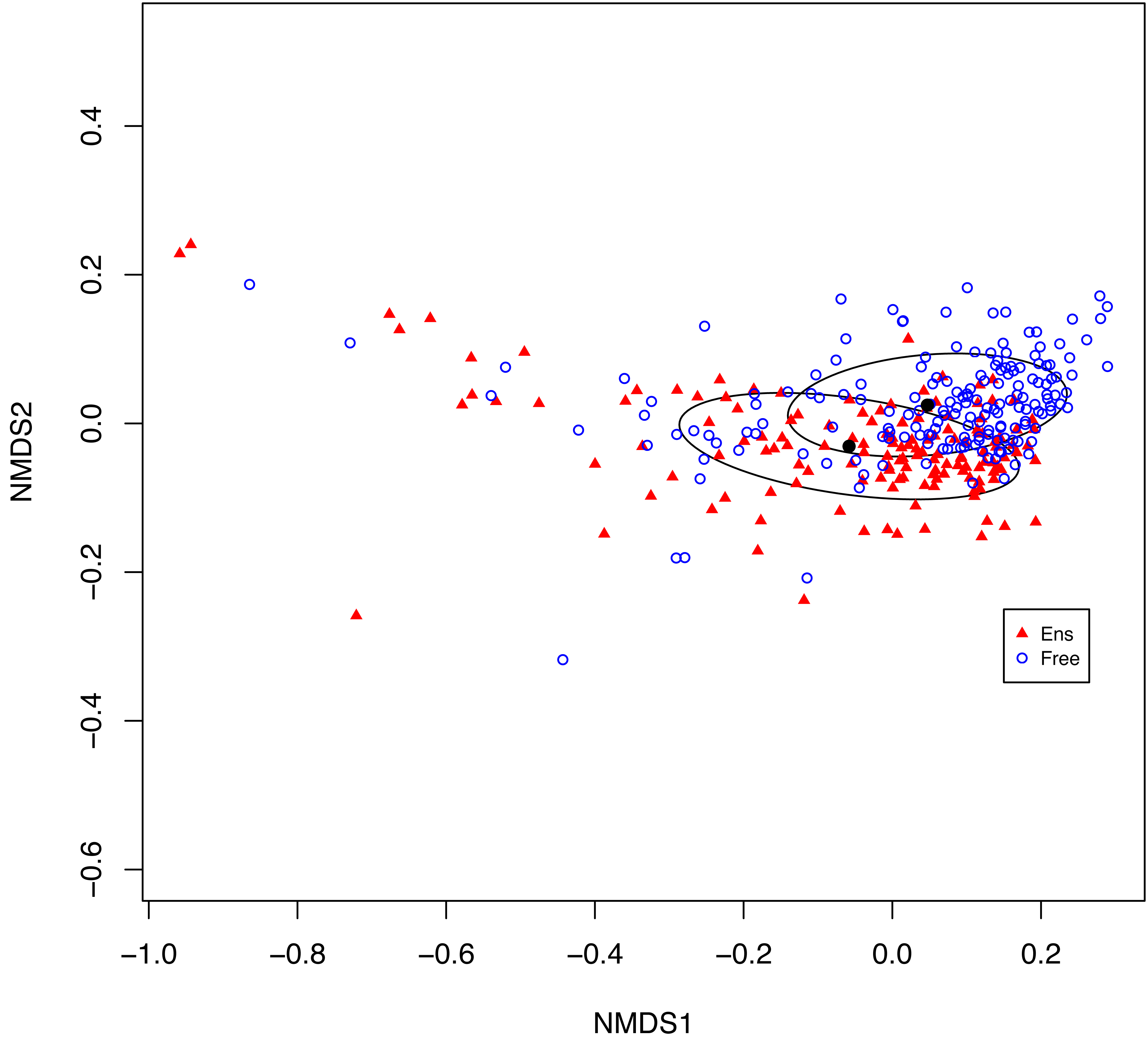

Supplement: S2 Fig — Plotted points are based on the relative proportions of 14 chemical peaks detected in cuticular hydrocarbon profiles of individual F. altipetens workers from enslaved colonies (Ens; red triangles) or free-living colonies (Free; blue circles). To illustrate clustering by either enslaved versus free-living status, standard deviation ellipses with centrioid points are plotted. (TIF) [file pone.0147498.s003.tif]
